# Human specific divergent cytoskeletal evolution defines genetic background for innate immune function

**DOI:** 10.1002/alz70859_098688

**Published:** 2025-12-25

**Authors:** Kinga Szigeti

**Affiliations:** ^1^ University at Buffalo, Buffalo, NY USA

## Abstract

**Background:**

Innate immune response is dependent on the ability of monocytes to infiltrate the tissue niches where pathogens reside, or tissue damage occurs. *CHRFAM7A* is a bi‐allelic uniquely human fusion gene between CHRNA7 and ULK4 present in 99.3% of humans. α7 nAChR has been implicated in innate immunity and as part of the cholinergic anti‐inflammatory response. The effect of *CHRFAM7A* alleles on innate immune function is unknown.

**Method:**

Isogenic iPSC derived microglia and monocytes are utilized as a model system and validated in human primary monocytes (N=100 donors). Actin and tubulin live imaging, migration and invasion assays are used to functionally characterize the alleles. Adaptive function to the mechanical properties of the tissue environment are studied on hydrogel models.

**Result:**

The translated direct allele leads to a hypomorphic α7 nicotinic acetylcholine receptor, while the inverted structural variant (SV) allele skews ULK4 short and long isoform ratio through genetic epistasis. The direct allele activates Rac1 and leads to a dynamic actin cytoskeleton and phenotypic switch to lamellipodia while the inverted allele acetylates a‐tubulin and stabilizes the microtubule cytoskeleton. Consequently, the alleles use distinct mechanisms for locomotion (direct‐invasion and inverted‐migration) and adaptation to hydrogel stiffness (direct‐lamellipodia and inverted‐polarization).

**Conclusion:**

*CHRFAM7A* infers divergent cytoskeletal gain of function leading to adaptation to tissue stiffness and chemotaxis. Importantly, the mechanism is not only human specific, but has a dichotomy with equal allele frequencies of the two *CHRFAM7A* alleles (direct and inverted) suggesting potentially profound translational significance. Understanding this bi‐allelic human genetic background builds the foundation for targeted therapeutic intervention for neuroinflammation.

